# The Clinical Characteristics and Prognosis Factors of Mild-Moderate Patients With COVID-19 in a Mobile Cabin Hospital: A Retrospective, Single-Center Study

**DOI:** 10.3389/fpubh.2020.00264

**Published:** 2020-06-05

**Authors:** Jishou Zhang, Menglong Wang, Mengmeng Zhao, Shanshan Guo, Yao Xu, Jing Ye, Wen Ding, Zhen Wang, Di Ye, Wei Pan, Menglin Liu, Dan Li, Zhen Luo, Jianfang Liu, Jun Wan

**Affiliations:** ^1^Department of Cardiology, Renmin Hospital of Wuhan University, Wuhan, China; ^2^Cardiovascular Research Institute, Renmin Hospital of Wuhan University, Wuhan, China; ^3^Hubei Key Laboratory of Cardiology, Wuhan, China; ^4^Department of Emergency, Renmin Hospital of Wuhan University, Wuhan, China; ^5^Department of Pediatrics, Renmin Hospital of Wuhan University, Wuhan, China

**Keywords:** novel mobile cabin hospitals, COVID-19, mild-moderate patients, clinical dynamics, prognosis

## Abstract

**Background:** Novel mobile cabin hospitals have been built to provide more makeshift beds for patients with COVID-19 in Wuhan. However, the characteristics of these patients needed be further described.

**Methods:** This was a retrospective, single-center study. A total of 869 patients with confirmed COVID-19 were admitted to Wuchang Mobile Cabin Hospital in Wuhan, between February 6th, 2020 and February 20th, 2020. The final date of follow-up was March 6th, 2020. Clinical characteristics and outcome data were collected and analyzed.

**Results:** Of 869 patients, the median age was 51 years (IQR, 40–58 years), and 377 patients (377/869; 43.4%) were men. A total of 616 patients (616/869; 70.9%) were discharged, 95 patients (95/869; 10.9%) were transferred to the designated hospital due to worsening condition (endpoint), and 158 patients (158/869; 18.2%) were still in the hospital. The incidence of the main symptoms, including fever, cough, fatigue, muscle aches, and anorexia, decreased with time. However, there were no differences in outcome among the patients with different onset times. Generally, both patients aged 45 years or older and patients with comorbidities were more likely to reach the endpoint (transfer to designated high-level hospitals due to condition worsen). In the other model, patients with the lung CT feature (e.g., ground-glass opacity, reticular/linear, air bronchogram, or consolidation shadow) were more likely to reach the endpoint.

**Conclusion:** Older age, comorbidity, special chest CT features (e.g., ground-glass opacity, reticular/linear, air bronchogram, or consolidation shadow) are associated with poor prognosis for mild-moderate patients. The initial symptoms of mild-moderate patients may become insidious, which deserves our attention.

## Introduction

In early 2020, coronavirus disease 2019 (COVID-19), which arises from severe acute respiratory syndrome coronavirus 2 (SARS-CoV-2) infection, has been the world's largest health crisis (1, 2). SARS-CoV-2 is rapidly spreading around the world and infected more than 4000,000 people worldwide as of May 16th 2020 (1, 3, 4). According to the coronavirus guidelines, the disease is generally classified into 4 types: mild, moderate, severe, and critical (5). Zhang reported that mild or moderate patients accounted for more than 80% of patients with COVID-19 (6). The severe and critical patients usually require more attention due to the poor outcomes, according to previous reports (7). However, the clinical characteristics and prognostic factors of mild-moderate patients have rarely been reported. In addition, these mild or moderate patients should be admitted to the hospital to prevent progression of the disease and should be isolated from susceptible populations to prevent further transmission. However, the limited capacity of designated hospitals for infectious disease patients makes the prevention and treatment of COVID-19 challenging.

Mobile cabin hospitals, generally composed of medical treatment units, ward units, technical support units, and others, are a type of modular health equipment with emergency treatment, surgical treatment, clinical testing, and other functions and are widely used in a variety of emergency treatment scenarios, the military field, and other fields (8). During the past infectious disease epidemics or natural disasters, mobile cabin hospitals have been put in place to cope with the shortage of medical sources (9, 10). However, the capacity of the hospital is limited. Compared to the traditional mobile hospitals, novel mobile cabin hospitals (also named as Fangcang hospitals in China) could provide many more medical beds in a short time. In Wuhan, several novel mobile cabin hospitals were transformed from large public facilities such as sports stadium and exhibition center in a very short time, providing thousands of beds to admit and treat mild-to-moderate COVID-19 patients. If condition worsening occurred, patients were transferred to a nearby designated hospital for critical patients.

In this study, we aimed to describe the clinical characteristics and outcomes of 869 hospitalized mild-moderate patients from Wuchang Mobile Cabin Hospital and compared the clinical findings of patients with COVID-19 stratified according to sex, age, comorbidity, and time of diseases onset.

## Methods

### Study Design and Participants

This was a retrospective study. The study involving human participants was reviewed and approved by the Institutional Ethics Board of Renmin Hospital of Wuhan University. Written informed consent from the participants' legal guardian/next of kin was not required to participate in this study in accordance with the national legislation and the institutional requirements. During the period of February 6th, 2020 to February 20th, 2020, 869 confirmed hospitalized cases of SARS-CoV2 admitted to Wuchang Mobile Cabin Hospital were enrolled in this study. The diagnosis of COVID-19 was made according to the Diagnosis and Treatment of Pneumonia Infected by Novel Coronavirus (5th trial edition) published by the General Office of the National Health Commission and the General Office of the National Administration of Traditional Chinese Medicine (11).

According to the Diagnosis and Treatment of Pneumonia Infected by Novel Coronavirus (5th trial edition), all of the recruited patients were classified as mild-moderate on admission. The detail criterion (11) for clinical classification: Mild: mild signs or symptoms, imaging shows no signs of pneumonia; Moderate: fever, respiratory tract symptoms, imaging shows pneumonia; Severe: satisfy any of the following: (1) respiratory distress, respiratory rate ≥30 beats per minute; (2) SpO_2_ ≤ 93% at resting; (3) arterial PaO_2_/FiO_2_ ≤ 300 mmHg; Critical: satisfy any of the following: (1) respiratory failure, need mechanical ventilation; (2) shock; (3) combined with other organ failure, requiring intensive care. The onset of disease was defined as the time of the first occurrence of related symptoms.

The outcome information of these patients was collected until March 6th, 2020, including remaining in the mobile cabin hospital, discharged, and transferred to the designated hospital for critical patients due to worsening of the patient's condition. The worsening of the condition of patients may have been due to COVID-19 or basic diseases. To be specific, if patients met any of the following criteria (12), they were quickly transferred to the designated higher-level hospitals: (1) met the criterion of severe or critical; (2) lung imaging showing a greater than 50% progression of lesions within 24–48 h; (3) development of basic disease, such as hypertension, diabetes, coronary artery disease, etc. To compare the outcomes of patients by stratification according to the onset time of diseases, we analyzed information within 15 days of admission.

### Data Collection

The clinical data (including basic information, clinical symptoms and signs, history, comorbidities, treatment and outcomes) were obtained by experienced clinicians based on the medical records system of the hospital. Manifestations on computed tomography (CT) were summarized by integrating the documentation or description in medical charts.

### Grouping According to Different Factors

Sex was classified as male or female. Since 99.7% of patients were less than 70 years, age was classified into three groups according to the population distribution: <45, 45–60, and >60 years. Comorbidities were determined based on the patient's self-report on admission and were initially treated as a categorical variable (yes vs. no). Onset time was classified into four groups based on the integration of time and population descriptions as follows: Period 1, January 16th to January 25th, 2020; Period 2, January 26th to January 31st, 2020; Period 3, February 1st to February 6th, 2020; and Period 4, February 7th to February 14th, 2020. Patients with unclear onset information were excluded from the onset time cohorts.

### Statistical Analysis

Statistical analyses were conducted with SPSS software version 22.0 (Chicago, USA) and EmpowerStats software. The continuous variables are expressed as the median and interquartile range (IQR), and the differences between any two groups were determined by the Mann-Whitney test. The categorical variables are presented as counts (percentages) and were compared with chi-square tests, although Fisher's exact test was used when the data were limited. The Kaplan-Meier test was used to compare the cumulative risk rate. Cox proportional hazard regression models and landmark analysis were applied to identify the potential risk factors associated with the endpoint as appropriate, with the hazard ratio (HR) and 95% confidence interval (95% CI) being reported.

## Results

### Clinical Characteristics and Outcomes of All Patients Treated in Novel Mobile Cabin Hospitals

Our database included 869 cases from Wuchang Mobile Cabin Hospital. Of these 869 cases, the median age was 52 years. A total of 377 patients (377/869; 43.4%) were males. A total of 121 patients (121/869; 13.9%) reported having at least one comorbidity, and 24 patients (24/869; 2.8%) had more than one comorbidity. The most common symptom was fever (565/869; 65%), followed by cough (424/869; 48.8%), fatigue (226/869; 26.0%), anorexia (216/869; 24.9%), and muscle aches (114/869; 13.1%). However, only 22 patients (22/839; 2.6%) had fever when vital signs were checked at admission, and the percent of patients showed the highest temperature > 37.3°C during hospital was only 7.2% (60/839), indicating that simple temperature screening may have limited effect in public. Ninety-one percent of patients showed a manifestation of pneumonia on lung CT, although some patients' lung CT scans were unavailable. The vast majority of patients (845/869; 97.2%) were treated with antiviral drugs, and all patients (100%) were treated with traditional Chinese medicine. Ultimately, 616 patients (616/869; 70.9%) were discharged from the novel mobile cabin hospital through March 6th, 2020. Ninety-five patients (95/869; 10.9%) reached the endpoint (transferred to the designated hospital for critical patients), and 158 patients (158/869; 18.2%) were still in the hospital. All data are shown in Table 1.

**Table 1:** Clinical characteristics and outcomes of total, male and female patients in mobile cabin hospital.

	**All (*n* = 869)**	**Male (*n* = 377)**	**Female (*n* = 492)**	***P*-value**
**Age, median (IQR), years**	51 (40, 58)	48 (38, 57)	52 (42, 59)	<0.001*
**Comorbidity, No. (%)**	121 (13.9)	53 (14.1)	68 (13.8)	0.920
Diabetes	21 (2.4)	7 (1.9)	14 (2.8)	0.347
Hypertension	91 (10.5)	45 (11.9)	46 (9.3)	0.217
Coronary heart disease	5 (0.6)	2 (0.5)	3 (0.6)	1
COPD/asthma	11 (1.3)	2 (0.5)	9 (1.8)	0.164
Cerebrovascular disease	1 (0.1)	0	1 (0.2)	1
Chronic renal disease	4 (0.5)	3 (0.8)	1 (0.2)	0.439
Chronic liver disease	5 (0.6)	2 (0.5)	3 (0.6)	1
Malignancy	6 (0.7)	1 (0.3)	5 (1.0)	0.362
Single comorbidity	97 (11.2)	44 (11.7)	53 (10.8)	0.677
≥2 comorbidities	24 (2.8)	9 (2.4)	15 (3.0)	0.555
**Symptoms, No. (%)**				
Fever	565 (65.0)	258 (68.4)	307 (62.4)	0.064
Symptoms of respiratory system	494 (56.8)	202 (53.6)	292 (59.3)	0.089
Sore throat	18 (2.1)	5 (1.3)	13 (2.6)	0.177
Cough	424 (48.8)	183 (48.5)	241 (49.0)	0.897
Expectoration	36 (4.1)	17 (4.5)	19 (3.9)	0.635
Chest tightness	104 (12.0)	25 (6.6)	79 (16.1)	<0.001*
Chest pain	13 (1.5)	5 (1.3)	8 (1.6)	0.718
Dyspnea	47 (5.4)	13 (3.4)	34 (6.9)	0.025*
Catarrhal symptoms	14 (1.6)	7 (1.9)	7 (1.4)	0.615
Symptoms of nervous and muscle system	258 (29.7)	120 (31.8)	138 (28.0)	0.227
Fatigue	226 (26.0)	113 (30.0)	113 (23.0)	0.02*
Dizziness	12 (1.4)	2 (0.5)	10 (2.0)	0.06
Headache	18 (2.1)	4 (1.1)	14 (2.8)	0.067
Muscle ache	114 (13.1)	70 (18.6)	44 (8.9)	<0.001*
Symptoms of alimentary system	269 (31.0)	135 (35.8)	134 (27.2)	0.007*
Anorexia	216 (24.9)	122 (32.4)	94 (19.1)	<0.001*
Nausea	18 (2.1)	5 (1.3)	13 (2.6)	0.177
Vomiting	16 (1.8)	5 (1.3)	11 (2.2)	0.323
Diarrhea	58 (6.7)	18 (4.8)	40 (8.1)	0.049*
**Signs on admission, median (IQR)**				
Temperature, °C	36.5 (36.3, 36.7)	36.5 (36.3, 36.7)	36.5 (36.3, 36.7)	
>37.3, No. (%)*^a^*	22 (2.6)	11 (3.1)	11 (2.3)	
Heart rate, bpm	81 (74, 90)	82 (74, 92)	80 (75, 89)	0.047*
Finger oxygen saturation, %	97 (96, 98)	97 (96, 98)	97 (96, 98)	0.237
Respiratory rate, bpm	20 (18, 21)	20 (18, 21)	20 (18, 20)	0.586
**Highest temperature during hospital**, **°C**				
>37.3, No. (%)*^a^*	60 (7.2)	33 (9.2)	27 (5.6)	0.06
**Characteristics of lung CT, No. (%)**^**b**^				
Pneumonia	424 (91.0)	173 (90.6)	251 (91.3)	0.796
Unilateral lung	50 (10.7)	22 (11.5)	28 (10.2)	0.647
Bilateral lung	374 (80.3)	151 (79.1)	223 (81.1)	0.588
Ground-glass opacity	339 (72.7)	147 (77.0)	192 (69.8)	0.088
Reticular/linear	220 (47.2)	101 (52.9)	119 (43.3)	0.041*
Air bronchogram	9 (1.9)	7 (3.7)	2 (0.7%)	0.054
Consolidation shadow	22 (4.7)	13 (6.8)	9 (3.3)	0.077
**Medical treatment, No. (%)**				
Antiviral treatment	845 (97.2)	365 (96.8)	480 (97.6)	0.507
Traditional Chinese medicine	869 (100)	377 (100)	492 (100)	1
**Outcome, No. (%)**				
Discharge from mobile cabin hospital	616 (70.9)	266 (70.6)	350 (71.1)	0.852
Transfer to the designated hospital	95 (10.9)	42 (11.1)	53 (10.8)	0.863
Staying in mobile cabin hospital	158 (18.2)	69 (18.3)	89 (18.1)	0.936

The total number of patients with available data: a: total = 839, male = 358, female = 481; b: total = 466, male = 191, female = 275. COPD, chronic obstructive pulmonary disease; IQR, interquartile range. P-values indicate differences between male and female patients. *indicates P-value <0.05.

### Clinical Characteristics According to Sex

Female patients were older than male patients (54 [44, 61] vs. 50 [39, 59], *p* < 0.001). Male patients were more likely to have fatigue (113/377, 30% vs. 113/492, 23%, *p* = 0.02), muscle aches (70/377, 18.6% vs. 44/4928.9%, *p* < 0.001), and anorexia (122/377, 32.4% vs. 94/492, 19.1%, *p* < 0.001). Female patients were more likely to have chest tightness (79/492, 16.1% vs. 25/377, 6.6%, *p* < 0.001), dyspnea (34/492, 6.9% vs. 13/377, 3.4%, *p* = 0.025), and diarrhea (40/492, 8.1% vs. 18/377, 4.8%, *p* = 0.049). Male patients were more likely to have reticular/linear manifestations (101/191, 52.9% vs. 119/275, 43.3%, *p* = 0.041) on lung CT, although no significant differences were observed in other features. The results may suggest that the conditions of male patients were possibly worse than those of females. However, there were no differences in outcomes between male patients and female patients. The data are shown in Table 1.

### Clinical Characteristics According to Age

Age was classified into three groups: <45 years (*n* = 323), 45–60 years (*n* = 378) and >60 years (*n* = 168). The group of older patients included fewer male patients than the group of younger patients (65/168, 38.7% vs. 144/378, 38.1% vs. 168/323, 52%). The group of older patients included more patients with at least one comorbidity (41/168, 24.4% vs. 64/378, 16.9% vs. 16/323, 5%), more patients with a single comorbidity (33/168, 19.6% vs. 50/378, 13.2% vs. 14/323, 4.3%) and more patients with two or more comorbidities (8/168, 4.8% vs. 14/378, 3.7% vs. 2/323, 0.6%). Conversely, fewer older patients had fever (101/168, 60.1% vs. 247/378, 65.3%, vs. 217/323, 67.2%) than younger patients, although the differences were not significant. In addition, older patients had lower heart rates and oxygen saturation on admission. CT results found that both the >60-year group and the 45- to 60-year group included more patients with pneumonia manifestations (97/99, 98% vs. 200/214, 93.5% vs. 127/153, 83%), which were more complex in the two older groups. Discharged patients were more commonly aged less than 45 than >60 years (244/323, 75.5% vs. 107/168, 63.7%). Both the >60-year group and the 45- to 60-year group had more patients (23/168, 13.7% vs. 51/378, 13.5% vs. 21/323, 6.5%) transferred to the designated hospital due to worsening condition, although significant differences between the >65-year group and the 45 to 60-year group were not observed. All data are shown in Table 2.

**Table 2:** Characteristics and outcomes of patients with different ages.

	**Age, years**		
	**<45 (*n* = 323)**	**45–60 (*n* = 378)**	**>60 (*n* = 168)**
**Sex, No. (%)**			
Male	168 (52.0)	144 (38.1)*	65 (38.7)*
Female	155 (48.0)	234 (61.9)	103 (61.3)
**Comorbidity, No. (%)**	16 (5.0)	64 (16.9)*	41 (24.4)*#
Diabetes	4 (1.2)	9 (2.4)	8 (4.8)*
Hypertension	10 (3.1)	52 (13.8)*	29 (17.3)*
Coronary heart disease	0	3 (0.8)	2 (1.2)
COPD/asthma	2 (0.6)	7 (1.9)	2 (1.2)
Cerebrovascular disease	0	0	1 (0.6)
Chronic renal disease	0	2 (0.5)	2 (1.2)
Chronic liver disease	1 (0.3)	3 (0.8)	1 (0.6)
Malignancy	1 (0.3)	2 (0.5)	3 (1.8)
Single comorbidity	14 (4.3)	50 (13.2)*	33 (19.6)*
≥2 comorbidities	2 (0.6)	14 (3.7)*	8 (4.8)*
**Symptoms, No. (%)**			
Fever	217 (67.2)	247 (65.3)	101 (60.1)
Symptoms of respiratory system	186 (57.6)	223 (59.0)	85 (50.6)
Sore throat	10 (3.1)	8 (2.1)	0*
Cough	161 (49.8)	186 (49.2)	77 (45.8)
Expectoration	16 (5.0)	16 (4.2)	4 (2.4)
Chest tightness	31 (9.6)	58 (15.3)*	15 (8.9)#
Chest pain	6 (1.9)	7 (1.9)	0
Dyspnea	12 (3.7)	23 (6.1)	12 (7.1)
Catarrhal symptoms	7 (2.2)	5 (1.3)	2 (1.2)
Symptoms of nervous and muscle system	89 (27.6)	110 (29.1)	59 (35.1)
Fatigue	77 (23.8)	97 (25.7)	52 (31.0)
Dizziness	6 (1.9)	5 (1.3)	1 (0.6)
Headache	7 (2.2)	7 (1.9)	4 (2.4)
Muscle ache	49 (15.2)	42 (11.1)	23 (13.7)
Symptoms of alimentary system	96 (29.7)	122 (32.3)	51 (30.4)
Anorexia	82 (25.4)	91 (24.1)	43 (25.6)
Nausea	3 (0.9)	12 (3.2)*	3 (1.8)
Vomiting	4 (1.2)	9 (2.4)	3 (1.8)
Diarrhea	20 (6.2)	28 (7.4)	10 (6.0)
**Signs on admission, median (IQR)**			
Temperature, °C	36.5 (36.3, 36.7)	36.5 (36.3, 36.7)	36.5 (36.3, 36.7)
Heart rate, bpm	82 (75, 92)	81 (75, 89)	80 (73, 89)
Finger oxygen saturation, %	97 (96, 98)	97 (96, 98)*	97 (96, 98)*
Respiratory rate, bpm	18 (20, 20)	18 (20, 20)	18 (20, 21)
**Highest temperature during hospital**, **°C**			
>37.3, No. (%)^a^	25 (8.0)	46 (12.6)*	12 (7.4)
**Characteristics of lung CT, No. (%)**^**b**^			
Pneumonia	127 (83.0)	200 (93.5)*	97 (98.0)*
Unilateral lung	20 (13.1)	21 (9.8)	9 (9.1)
Bilateral lung	107 (69.9)	179 (83.6)*	88 (88.9)*
Ground-glass opacity	101 (66.0)	163 (76.2)*	75 (75.8)
Reticular/linear	51 (33.3)	109 (50.9)*	60 (60.6)*
Air bronchogram	2 (1.3)	6 (2.8)	1 (1.0)
Consolidation shadow	4 (2.6)	15 (7.0)	3 (3.0)
**Medical treatment, No. (%)**			
Antiviral treatment	314 (97.2)	372 (98.4)	160 (95.2)
Traditional Chinese medicine	323 (100)	378 (100)	168 (100)
**Outcome, No. (%)**			
Discharge from mobile cabin hospital	244 (75.5)	265 (70.1)	107 (63.7)*
Transfer to the designated hospital	21 (6.5)	51 (13.5)*	23 (13.7)*
Staying in mobile cabin hospital	58 (18.0)	62 (16.4)	38 (22.6)

99.7% of patients were less than 70 years. The total number of patients with available data: a: <45 years = 313, 45–60 years = 364, >60 years = 162; b: <45 years = 153, 45–60 years = 214, >60 years = 99. COPD, chronic obstructive pulmonary disease; IQR, interquartile range. patients. *p < 0.05 vs. <45 years group. ^#^p < 0.05 vs. 45–60 years group.

### Clinical Characteristics According to Comorbidity

Of the 869 cases, 121 patients (121/869; 13.9%) were reported to have at least one comorbidity. The most common comorbidities included hypertension (91/869; 10.5%), diabetes (21/869; 2.4%) and COPD/asthma (11/869; 1.6%) (Table 1). Patients with at least one comorbidity were older (median: 59 vs. 51 years, *p* < 0.001). Of the patients who were transferred to the designated hospital for further treatment, there were more patients with at least one comorbidity than without (23/121, 19% vs. 72/748, 9.6%, *p* = 0.002). No significant differences were observed in the discharged patients between the two groups (Table 3).

**Table 3:** Characteristics and outcomes of patients with or without any comorbidity.

	**Any comorbidity**		
	**No (*n* = 748)**	**Yes (*n* = 121)**	***P*-value**
**Age, median (IQR), years**	49 (39, 58)	57 (51, 62)	<0.001*
**Sex, No. (%)**			
Male	324 (43.3)	53 (43.8)	0.920
Female	424 (56.7)	68 (56.2)	
**Symptoms, No. (%)**			
Fever	481 (64.3)	84 (69.4)	0.274
Symptoms of respiratory system	416 (55.6)	78 (64.5)	0.068
Sore throat	18 (2.4)	0	0.167
Cough	359 (48.0)	65 (53.7)	0.243
Expectoration	31 (4.1)	5 (4.1)	0.995
Chest tightness	89 (11.9)	15 (12.4)	0.876
Chest pain	11 (1.5)	2 (1.7)	1
Dyspnea	38 (5.1)	9 (7.4)	0.287
Catarrhal symptoms	10 (1.3)	4 (3.3)	0.227
Symptoms of nervous and muscle system	218 (29.1)	40 (33.1)	0.382
Fatigue	192 (25.7)	34 (28.1)	0.572
Dizziness	10 (1.3)	2 (1.7)	1
Headache	17 (2.3)	1 (0.8)	0.443
Muscle ache	102 (13.6)	12 (9.9)	0.261
Symptoms of alimentary system	237 (31.7)	32 (26.4)	0.248
Anorexia	193 (25.8)	23 (19.0)	0.109
Nausea	15 (2.0)	3 (2.5)	1
Vomiting	15 (2.0)	1 (0.8)	0.596
Diarrhea	47 (6.3)	11 (9.1)	0.251
**Signs on admission, median (IQR)**			
Temperature, °C	36.5 (36.3, 36.7)	36.5 (36.3, 36.7)	0.286
Heart rate, bpm	81 (75, 90)	79 (73, 88)	0.177
Finger oxygen saturation, %	97 (96, 98)	97 (96, 98)	0.435
Respiratory rate, bpm	20 (18, 21)	20 (18, 21)	0.904
**Highest temperature during hospital, median (IQR)**, **°C**	36.9 (36.8, 37)	36.9 (36.8, 37.1)	0.677
**Characteristics of lung CT, No. (%)***^***a***^*			
Pneumonia	364 (90.5)	60 (93.8)	0.406
Unilateral lung	37 (9.2)	13 (20.3)	0.008*
Bilateral lung	327 (81.3)	47 (73.4)	0.14
Ground-glass opacity	290 (72.1)	49 (76.6)	0.46
Reticular/linear	185 (46.0)	35 (54.7)	0.197
Air bronchogram	7 (1.7)	2 (3.1)	0.796
Consolidation shadow	18 (4.5)	4 (6.3)	0.761
**Medical treatment, No. (%)**			
Antiviral treatment	725 (96.9)	120 (99.2)	0.271
Traditional Chinese medicine	748 (100)	121 (100)	1
**Outcome, No. (%)**			
Discharge from mobile cabin hospital	533 (71.3)	83 (68.6)	0.55
Transfer to the designated hospital	72 (9.6)	23 (19.0)	0.002*
Staying in mobile cabin hospital	143 (19.1)	15 (12.4)	0.075

The total number of patients with available data: a: patients with comorbidities = 402, patients without = 64. COPD, chronic obstructive pulmonary disease; IQR, interquartile range. P-values indicate differences between patients with and without comorbidities. *indicates P-value < 0.05.

### Clinical Characteristics According to Onset Time of COVID-19

**Figure 1:**
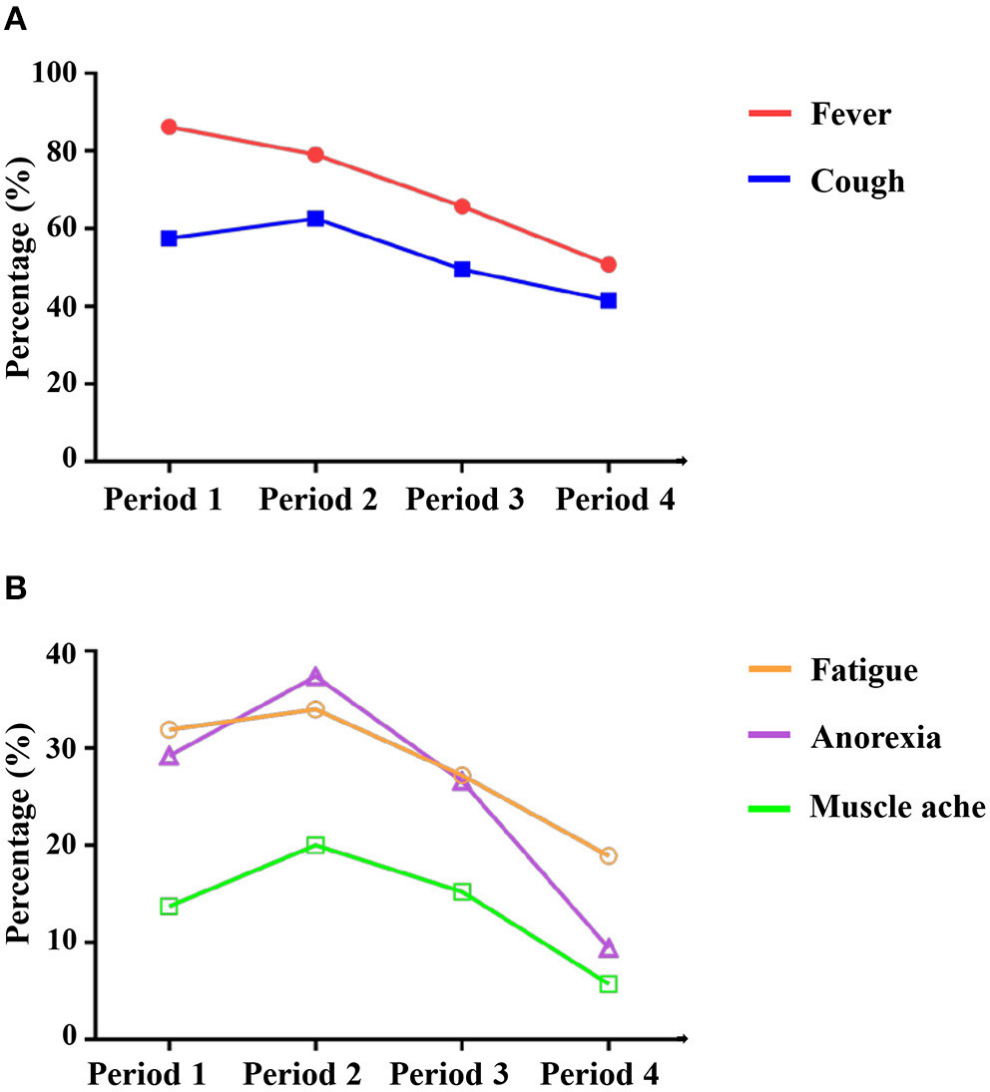
Typical clinical symptoms of patient with COVID-19 showed a decreasing trend with time. X-axis indicates onset time of the disease. **(A)** fever and cough; **(B)** fatigue, anorexia, and muscle ache.

The onset time cohorts were classified into four groups: Period 1, January 16th to January 25th, 2020 (*n* = 226); Period 2, January 26th to January 31st, 2020 (*n* = 235); Period 3, February 1st to February 6th, 2020 (*n* = 184); and Period 4, February 7th to February 14th, 2020 (*n* = 106). The incidence of the main symptoms, including fever (195/226, 86.3% vs. 186/235, 79.1% vs. 121/184, 65.8% vs. 54/106, 50.9%), cough (130/226, 57.5% vs. 147/235, 62.6% vs. 91/184, 49.5% vs. 44/106, 41.5%), fatigue (72/226, 31.9% vs. 80/235, 34% vs. 50/184, 27.2% vs. 20/106, 18.9%), muscle aches (31/226, 13.7% vs. 47/235, 20% vs. 28/184, 15.2% vs. 6/106, 5.7%), and anorexia (66/226, 29.2% vs. 88/235, 37.4% vs. 49/184, 26.6% vs. 10/106, 9.4%), decreased with time (Figures 1A,B). The analyses of lung CT found that the period 4 group had fewer patients with pneumonia. The severity of CT manifestations was also decreasing, which was illustrated by the following items: rate of unilateral lung (5/123, 4.1% vs. 14/137, 10.2% vs. 16/106, 15.1% vs. 12/58, 20.7%), rate of bilateral lung (108/123, 87.8% vs. 118/137, 86.1% vs. 82/106, 77.4% vs. 36/58, 62.1%), and rate of ground-glass opacity (92/123, 74.8% vs. 106/137, 77.4% vs. 80/106, 75.5% vs. 36/58, 62.1%). However, there were no differences in the outcomes among the four groups within 15 days of admission. The data are shown in Table 4.

**Table 4:** Characteristics and outcomes of patients with different onset times.

	**Period 1 (*n* = 226)**	**Period 2 (*n* = 235)**	**Period 3 (*n* = 184)**	**Period 4 (*n* = 106)**
**Age, No. (IQR), years**	52 (40, 60)	50 (39, 58)	52 (42, 59)	46 (38, 56)*&
**Sex, No. (%)**				
Male	97 (42.9)	89 (37.9)	85 (46.2)	49 (46.2)
Female	129 (57.1)	146 (62.1)	99 (53.8)	57 (53.8)
**Comorbidity, No. (%)**	32 (14.2)	36 (15.3)	28 (15.2)	20 (18.9)
Diabetes	8 (3.5)	7 (3.0)	3 (1.6)	1 (0.9)
Hypertension	22 (9.7)	31 (13.2)	19 (10.3)	15 (14.2)
Coronary heart disease	1 (0.4)	1 (0.4)	1 (0.5)	2 (1.9)
COPD/asthma	3 (1.3)	3 (1.3)	4 (2.2)	1 (0.9)
Chronic renal disease	1 (0.4)	1 (0.4)	1 (0.5)	1 (0.9)
Chronic liver disease	2 (0.9)	1 (0.4)	0	1 (0.9)
Malignancy	1 (0.4)	1 (0.4)	3 (1.6)	1 (0.9)
Only one comorbidity	35 (15.5)	27 (11.5)	24 (13.0)	18 (17.0)
≥2 comorbidity	7 (3.1)	9 (3.8)	4 (2.2)	2 (1.9)
**Symptoms, No. (%)**				
Fever	195 (86.3)	186 (79.1)*	121 (65.8)*#	54 (50.9)*#&
Symptoms of respiratory system	150 (66.4)	161 (68.5)	108 (58.7)#	60 (56.6)#
Sore throat	5 (2.2)	4 (1.7)	3 (1.6)	5 (4.7)
Cough	130 (57.5)	147 (62.6)	91 (49.5)#	44 (41.5)*#
Expectoration	14 (6.2)	7 (3.0)	9 (4.9)	5 (4.7)
Chest tightness	35 (15.5)	32 (13.6)	18 (9.8)	17 (16.0)
Chest pain	3 (1.3)	0	5 (2.7)#	4 (3.8)#
Dyspnea	14 (6.2)	15 (6.4)	12 (6.5)	6 (5.7)
Catarrhal symptoms	2 (0.9)	4 (1.7)	3 (1.6)	4 (3.8)
Symptoms of nervous and muscle system	81 (35.8)	90 (38.3)	56 (30.4)	27 (25.5)#
Fatigue	72 (31.9)	80 (34.0)	50 (27.2)	20 (18.9)*#
Dizziness	3 (1.3)	4 (1.7)	0	4 (3.8)&
Headache	7 (3.1)	4 (1.7)	5 (2.7)	2 (1.9)
Muscle ache	31 (13.7)	47 (20.0)	28 (15.2)	6 (5.7)*#&
Symptoms of alimentary system	79 (35.0)	107 (45.5)*	62 (33.7)#	18 (17.0)*#&
Anorexia	66 (29.2)	88 (37.4)	49 (26.6)#	10 (9.4)*#&
Nausea	4 (1.8)	8 (3.4)	4 (2.2)	2 (1.9)
Vomiting	5 (2.2)	7 (3.0)	3 (1.6)	1 (0.9)
Diarrhea	14 (6.2)	24 (10.2)	13 (7.1)	6 (5.7)
**Signs on admission, median (IQR)**				
Temperature, °C	36.5 (36.3, 36.7)	36.5 (36.3, 36.7)	36.5 (36.4, 36.7)	36.5 (36.3, 36.6)&
Heart rate, bpm	81 (74, 90)	81 (75, 90)	79 (72, 89)	85 (76, 90)&
Finger oxygen saturation, %	97 (96, 98)	97 (96, 98)	97 (95, 98)#	97 (96, 98)&
Respiratory rate, bpm	20 (18, 21)	20 (18, 21)	20 (18, 20)	20 (19, 21)
**Characteristics of lung CT, No. (%)***^***a***^*				
Pneumonia	113 (91.9)	132 (96.4)	98 (92.5)	48 (82.8)#
Unilateral lung	5 (4.1)	14 (10.2)	16 (15.1)*	12 (20.7)*#
Bilateral lung	108 (87.8)	118 (86.1)	82 (77.4)*	36 (62.1)*#&
Ground-glass opacity	92 (74.8)	106 (77.4)	80 (75.5)	36 (62.1)#
Reticular/linear	57 (46.3)	63 (46.0)	52 (49.1)	23 (39.7)
Air bronchogram	1 (0.8)	3 (2.2)	1 (0.9)	1 (1.7)
Consolidation shadow	3 (2.4)	4 (2.9)	7 (6.6)	1 (1.7)
**Medical treatment, No. (%)**				
Antiviral treatment	223 (98.7)	233 (99.1)	181 (98.4)	102 (96.2)
Traditional chinese medicine	226 (100)	235 (100)	184 (100)	106 (100)
**Outcome within 15 days after admission, No. (%)**				
Discharge from mobile cabin hospital	110 (48.7)	122 (51.9)	82 (44.6)	49 (46.2)
Transfer to the designated hospital	12 (5.3)	7 (3.0)	10 (5.4)	6 (5.7)
Staying in mobile cabin hospital	104 (46.0)	106 (45.1)	92 (50.0)	51 (48.1)

Period 1: January 16th to January 25th, 2020; Period 2: January 26th to January 31st, 2020; Period 3: February 1st to February 6th, 2020; Period 4: February 7th to February 14th, 2020. The total number of patients with available data: a: Period 1 = 123, Period 2 = 137, Period 3 = 106, Period 4 = 58. COPD, chronic obstructive pulmonary disease; IQR, interquartile range. *p < 0.05 vs. Period 1; ^#^p < 0.05 vs. Period 2; ^&^p < 0.05 vs. Period 3.

### Prognostic Analyses

**Figure 2:**
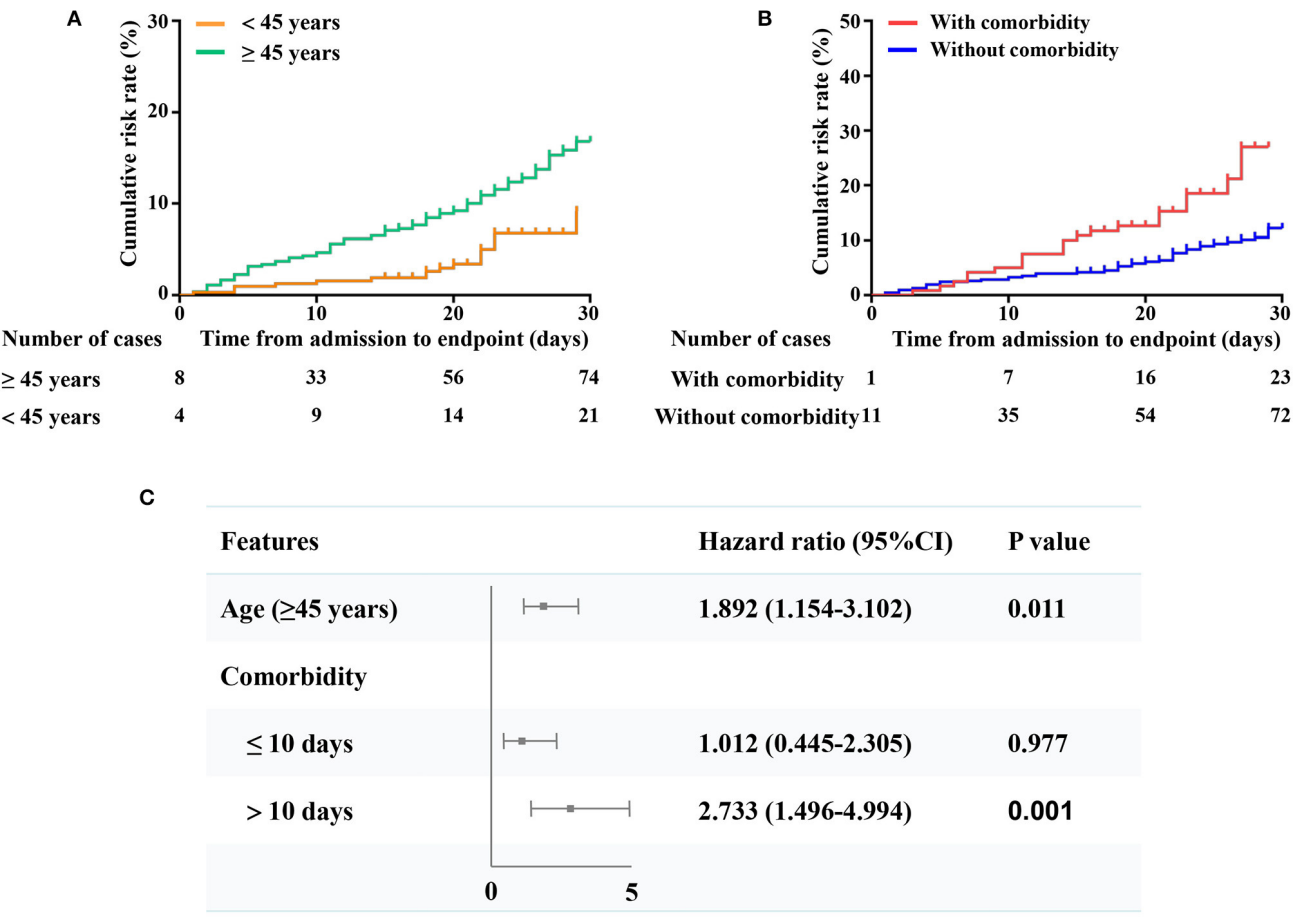
Comparison of the time-dependent risks and predictors of the endpoint. **(A)** The time-dependent risk of reaching to the endpoint between patients aged 45 or older (bottle green curve) and patients of less than 45 years (orange curve); **(B)** The time-dependent risk of reaching to the composite endpoints between patients with (red curve) or without any comorbidity (dark blue curve); **(C)** Shown in the figure are the hazards ratio (HR) and the 95% confidence interval (95% CI) for the risk factors associated with the endpoint (transferred to the designated hospitals for critical patients due to condition worsen). The scale bar indicates the HR. The model of age has been adjusted with gender and comorbidity. The model of comorbidity has been adjusted with gender and age.

We defined transfer to a designated hospital as the endpoint. Patients aged 45 or older had significantly higher risks of reaching the endpoint than those younger than 45 years old (*p* < 0.05) (Figure 2A). Patients with at least one comorbidity also had significantly higher risks of reaching the endpoint than those without comorbidities (*p* < 0.05) (Figure 2B). After adjusting for comorbidities and sex, patients aged 45 or older were more likely to reach the endpoint than those younger than 45 years old (HR, 1.892, 95% CI, 1.154–3.102, *p* = 0.011) (Figure 2C). After adjusting for age and sex, patients with comorbidities were more likely to reach the endpoint than those without comorbidities (HR, 2.733, 95% CI, 1.496–4.994, *p* = 0.001) (Figure 2C) when staying in the mobile cabin hospital for more than 10 days. However, there were no significant differences between patients with or without comorbidities within 10 days of hospitalization (Figure 2C).

Since a total of 466 patients were available in lung CT reports. Therefore, we recruited these 466 patients in a new model to analyze the CT findings of prognostic value. The univariate proportion COX regress analysis found that several lung CT features (ground-glass opacity, reticular/linear, air bronchogram, and consolidation shadow) were associated with prognosis. The multivariate COX regression analysis indicated that ground-glass opacity (HR, 2.096, 95% CI, 1.102–3.985, *p* = 0.024), reticular/linear (HR, 2.07, 95% CI, 1.275–3.362, *p* = 0.003), air bronchogram (HR, 4.741, 95% CI, 1.869–12.029, *p* = 0.001), and consolidation shadow (HR, 8.994, 95% CI, 4.953–16.331, *p* < 0.001) were associated with the poor outcome (transfer to the designated high-level hospitals due to condition worsen) for mild-moderate COVID-19 patients. The multivariate COX regression model has adjusted age, sex, and comorbidity (Table 5).

**Table 5:** The effect of chest CT on the prognosis of mild-moderate COVID-19 patients was analyzed by preparation COX regression.

**Feature**	**HR**	**95% CI**	***P*-value**
**Model 1**			
Pneumonia	3.393	0.833–13.828	0.088
Bilateral lung	1.523	0.783–2.964	0.215
Ground-glass opacity	2.266	1.196–4.291	0.012*
Reticular/linear	2.272	1,417–3.644	0.001*
Air bronchogram	5.807	2.338–14.421	<0.001*
Consolidation shadow	8.971	4.997–16.108	<0.001*
**Model 2**			
Ground-glass opacity	2.096	1.102–3.985	0.024*
Reticular/linear	2.07	1.275–3.362	0.003*
Air bronchogram	4.741	1.869–12.029	0.001*
Consolidation shadow	8.994	4.953–16.331	<0.001*

466 patients were enrolled in model 1 and model 2, since a total of 466 patients were available on CT reports. HR, hazard ratio. *indicates p-value <0.05.

*Model 1: univariate preparation COX regression*.

*Model 2: multivariate preparation COX regression: Sex, age, and comorbidity were adjusted*.

## Discussion

Our study was designed to analyze the clinical findings and outcomes of mild-moderate patients in one novel mobile cabin hospital in Wuhan, China. Our study found that older age, comorbidities, several lung CT features (e.g., ground-glass opacity, reticular/linear, air bronchogram, or consolidation shadow) were associated with aggravation of patients' conditions, which indicated that patients with these characteristics should receive additional attention in mobile cabin hospitals. In addition, typical clinical symptoms showed a decreasing trend with time, which suggested that the initial symptoms of mild-moderate patients with COVID-19 may become insidious.

Several articles related to COVID-19 have revealed that most of the patients infected with SARS-CoV-2 had mild or moderate infections (2, 6, 13). These patients with mild-moderate disease could recover with general treatment, although a few patients require intensive care due to the worsening of their condition (9). However, these patients were not effectively treated or isolated due to limited medical resources in the early stages. The activities of these mild-moderate patients further aggravated the spread of the disease in the community. Therefore, several novel mobile hospitals were built to address this difficulty. From February 6th 2020 to February 20th, 869 mild-moderate patients were admitted to one of these hospitals, Wuchang Fangcang Hospital that was built within 3 days and could offer 800 beds for patients. Of the 869 patients, 70.9% recovered in the hospital, and 10.9% were transferred to the high-level designated hospital due to patients' condition aggravation in a timely manner for further treatment. Literature has demonstrated that cabin hospitals played a critical role in the management of previous infectious disease and disasters (9, 10). Compared to the traditional mobile hospitals, these novel cabin hospitals had three key characteristics (rapid construction, massive scale, and low cost) and five essential functions (isolation, triage, basic medical care, frequent monitoring and rapid referral, and essential living and social engagement) (14). The earlier implementation of social distancing could obviously limit the epidemic and even reduce death (15). Interestingly, 12 days after the first fangcang hospitals started admitting patients, the number of confirmed cases in Wuhan steadily declined from Feb 18th, 2020 (16). Another study also suggested that these novel mobile cabin hospitals were characterized by flexibility and played an important role in the control of epidemic (17). Therefore, fangcang hospitals may provide inspires for other countries in COVID-19 epidemic.

Sex differences among patients infected with SARS-CoV-2 have been described (18). In our study, sex differences existed in the symptoms of COVID-19. Females had more chest tightness, dyspnea and diarrhea, and male patients had a higher rate of fatigue, muscle aches, and anorexia. However, the prognosis was not influenced by sex, although a previous study suggested that males were associated with more severe cases. This may be because patients in the hospital had mild disease on admission.

Several studies have suggested that older age and comorbidities are significantly associated with composite endpoints or death (2, 19, 20). Chest CT plays a critical role in the diagnosis and evaluation of COVID-19. The incidences of consolidation, linear opacities, crazy-paving pattern, and bronchial wall thickening in severe/critical patients were significantly higher than those of the ordinary patients (21). Our study found that relatively severe CT manifestations existed in older age patients or patients with at least one comorbidity. More patients aged 45 years or older reached the endpoint, and older age was a potential risk factor for the endpoint. However, the patients over 60 years did not have more cumulative risk than the 45–60 years group, which was not consistent with the literature reports (22, 23). This may be due to the age limitation of the mobile hospital that required advanced age patients, especially those with comorbidities, to be directly admitted to designated hospitals. Similarly, comorbidities may be another risk factor for the development of endpoints. In the other proportion COX regression model, results showed that several chest CT features (e.g., ground-glass opacity, reticular/linear, air bronchogram, or consolidation shadow) were associated with the poor outcome for mild-moderate COVID-19 patients. These results reminded us that patients with these risk factors should receive more attention to prevent patients' condition aggravation. Or, the doctors should identify these patients early and transfer them to the high-level designated hospitals.

The characteristics of generational transmission may be diverse due to virus mutations. The initial symptoms of infected patients may have changed with time. One study suggested that a novel SARS-CoV-2 mutation (ORF3a) had been found in Europe and may appear to be spreading worldwide (24). Therefore, we explored the clinical dynamics in mild-moderate patients from Wuchang Mobile Cabin Hospital. Our results revealed that the typical symptoms of COVID-19, including fever, cough, fatigue, muscle aches, and anorexia, showed a decreasing trend with time. In addition, catarrhal symptoms showed an increasing trend, although the difference was not significant. The results suggested that the typical symptoms of mild-moderate patients may become insidious, especially in the later stage of epidemic. Several reasons may contribute to the trend. First, as previously described, virus mutation may be responsible for the phenomenon, although the evidence is limited. Secondly, the detection capability of SARS-CoV-2 for contacts were furtherly enhanced, which lead to the recognition of COVID-19 patients before the symptoms appeared (15). Besides, the number of new cases was gradually reduced after Feb 2nd, 2020 and the medical assistance measures were gradually boosted during this period, which gave hospitals extra capacity to deal with patients with mild symptoms or single symptoms (14, 15). In brief, while some factors may affect the registration of patient's symptoms, it may be hard for health workers to identify COVID-19 according to the symptoms. In addition, the conditions on lung CT showed an improving trend, although the outcomes of patients within 15 days of admission were not different among the groups.

Some limitations existed in our study. Laboratory tests of most patients were unavailable due to the limitations of converted hospitals. Another limitation of our study was the self-report of comorbidities on admission. In addition, only 869 patients from one mobile cabin hospital were analyzed. A larger sample size may further increase the reliability of the conclusion.

## Conclusion

Older age, comorbidities and some chest CT features (e.g., ground-glass opacity, reticular/linear, air bronchogram, or consolidation shadow) were associated with poor outcomes for these mild-moderate patients. The initial symptoms of mild-moderate patients with COVID-19 may became insidious and deserve our attention.

## Data Availability Statement

The raw data supporting the conclusions of this article will be made available by the authors, without undue reservation.

## Ethics Statement

The studies involving human participants were reviewed and approved by the Institutional Ethics Board of Renmin Hospital of Wuhan University. Written informed consent from the participants' legal guardian/next of kin was not required to participate in this study in accordance with the national legislation and the institutional requirements.

## Author Contributions

MW and SG had full access to all of the data in the study. JZ, MW, and MZ designed the study and analysis the data. JZ wrote the manuscript. JZ, MW, MZ, and SG contributed equally to the study. JY, DY, ZW, WD, DL, ML, JL, WP, ZL, YX, and MZ critical revision of the manuscript for important intellectual content. MW and JW study supervision.

## Conflict of Interest

The authors declare that the research was conducted in the absence of any commercial or financial relationships that could be construed as a potential conflict of interest.
